# Neuronal Pre- and Postconditioning via Toll-like Receptor 3 Agonist or Extracorporeal Shock Wave Therapy as New Treatment Strategies for Spinal Cord Ischemia: An In Vitro Study

**DOI:** 10.3390/jcm11082115

**Published:** 2022-04-11

**Authors:** Daniela Lobenwein, Rosalie Huber, Lars Kerbler, Alexandra Gratl, Sabine Wipper, Can Gollmann-Tepeköylü, Johannes Holfeld

**Affiliations:** 1 Department of Vascular Surgery, Medical University of Innsbruck, 6020 Innsbruck, Austria; alexandra.gratl@i-med.ac.at (A.G.); sabine.wipper@i-med.ac.at (S.W.); 2 Department of Cardiac Surgery, Medical University of Innsbruck, 6020 Innsbruck, Austria; rosalie.huber@kh-schwaz.at (R.H.); can.gt@i-med.ac.at (C.G.-T.); johannes.holfeld@i-med.ac.at (J.H.); 3 Department of Anesthesiology and Intensive Care, Medical University of Innsbruck, 6020 Innsbruck, Austria; lars.kerbler@i-med.ac.at

**Keywords:** thoracoabdominal aneurysm, spinal cord ischemia, ischemic conditioning, Toll-like receptor 3, shock wave therapy

## Abstract

Spinal cord ischemia (SCI) is a devastating and unpredictable complication of thoracoabdominal aortic repair. Postischemic Toll-like receptor 3 (TLR3) activation through either direct agonists or shock wave therapy (SWT) has been previously shown to ameliorate damage in SCI models. Whether the same applies for pre- or postconditioning remains unclear. In a model of cultured SHSY-5Y cells, preconditioning with either poly(I:C), a TLR3 agonist, or SWT was performed before induction of hypoxia, whereas postconditioning treatment was performed after termination of hypoxia. We measured cytokine expression via RT-PCR and utilized Western blot analysis for the analysis of signaling and apoptosis. TLR3 activation via poly(I:C) significantly reduced apoptotic markers in both pre- and postconditioning, the former yielding more favorable results through an additional suppression of TLR4 and its downstream signaling. On the contrary, SWT showed slightly more favorable effects in the setting of postconditioning with significantly reduced markers of apoptosis. Pre- and post-ischemic direct TLR3 activation as well as post-ischemic SWT can decrease apoptosis and proinflammatory cytokine expression significantly in vitro and might therefore pose possible new treatment strategies for ischemic spinal cord injury.

## 1. Introduction

The surgical, endovascular, and medical treatment of thoracoabdominal aortic aneurysm (TAAA) poses a great challenge mainly due to a multitude of affected organ systems [1,2]. As the majority of TAAAs subsist asymptomatically, their detection often results from incidental findings [3]. Hence, definitive occurrences, i.e., incidence and prevalence, of TAAAs remain unclear to date but are estimated to account for around 10% of all detected aortic aneurysms [4]. As a result of progressively aging communities, advances in medicine, and an ever-increasing number of conducted radiological studies, the prevalence of TAAAs is expected to grow, leading to a higher demand for aortic repair [5].

**Figure 1:**
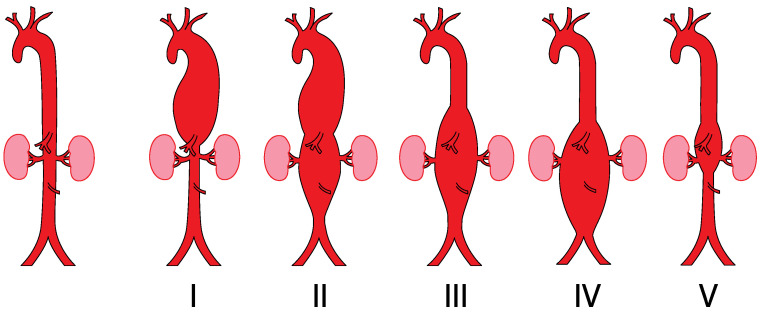
Crawford classification of thoracoabdominal aortic aneurysms (TAAAs) modified according to Safi [7]. Rates of spinal cord ischemia (SCI) vary between 6.3% (class II) and 1.4% (class IV) after surgical or endovascular aortic repair.

Based on their anatomical location and extent, TAAAs can be differentiated in accordance with the Crawford classification, which defines five different types of TAAA [6,7] (Figure 1). Type II describes the most spatially extensive group of TAAAs, which extends from the left subclavian artery down to the aortoiliac bifurcation [6] (Figure 1). Accordingly, these type II TAAAs pose the highest risk of end-organ ischemia during treatment [6,8].

For TAAA repair, open and endovascular techniques are available, and individual strategies depend on anatomical as well as patient-related factors [3]. All therapeutic options bear the risk of end-organ ischemia, which encompasses renal, mesenterial, or spinal cord ischemia [1,2,9]. Its risk is mainly determined by the pathological features and spatial extent of the individual aneurysm [6].

Blood is supplied to the spinal cord by the anterior spinal artery, which is fed by the vertebral arteries as well as the posterior spinal and segmental arteries [10]. The artery of Adamkiewicz—also known as arteria radicularis magna or great anterior radiculomedullary artery, which provides the major blood supply of the caudal cord in most individuals—typically originates from the left side of the aorta between T8 and L2 [11]. Moreover, collaterals to the spinal cord can also derive from the internal iliac arteries, sacral arteries, and from the inferior mesenteric artery [10]. Therefore, the treatment of TAAAs in this location, either by open surgical interposition of a prosthetic graft or endovascular coverage, poses a major risk for spinal cord ischemia [9]. Apart from Crawford type II graduation, the necessity of emergency or extensive aortic repair, left subclavian artery coverage, prior aortic repair, the amount of sacrificed segmental arteries, prolonged aortic clamping, vascular steal syndrome, advanced patient age, renal dysfunction, systemic hypotension, and acute anemia add up as risk factors for the development of spinal cord ischemia [9,12,13,14].

Depending on the affected segments of the spinal cord, clinical manifestations of spinal cord ischemia range from sensory to motor neurological deficits, including lower extremity paraparesis and paraplegia, and autonomic dysregulation [15]. Temporally, spinal cord ischemia and its clinical effects can be classified into immediate/acute and delayed types [13]. While acute type SCI usually occurs within hours after surgical or endovascular aortic repair, the latter manifests up to 91 days postoperatively [13,15,16]. It is hypothesized that acute-onset spinal cord ischemia is mainly related to immediate hypoxic neuronal cell death and ischemia-reperfusion injury, whereas the delayed type correlates with postischemic chronic inflammation, causing additional neurodegeneration [17].

The incidences of spinal cord ischemia following open vs. endovascular aortic repair differ [12]. After open aortic surgery, the incidence of postoperative paraplegia or paraparesis varies between 2.3% and 16% but rises significantly (up to 40%) in emergency repair situations [8,12,18,19]. On the contrary, the incidence of postoperative SCI is lower in thoracic endovascular aortic repair (TEVAR) and accounts for 3–5% of procedures [19] but reaches up to 17.7% in fenestrated and branched endovascular aortic repair (fEVAR and bEVAR) procedures [20]. However, due now to the more frequent use of endovascular repair of TAAAs, time will tell whether an increase in performed endovascular procedures will be paralleled by an absolute increase in SCI following endovascular aortic repair.

Rates of SCI also differ according to their Crawford classification: while 3.3% of patients suffering from Crawford type I aneurysm developed postoperative SCI, 6.3% of type II, 2.6% of type III, and 1.4% of type IV thoracoabdominal aneurysms were affected [8,12]. In order to relieve the burden of spinal cord ischemia (SCI), preventive interventions have been developed in past years. These include hypothermic circulatory arrest, reimplantation of intercostal arteries in open aortic repair, brief aortic clamping and short duration of left heart bypass, as well as spinal cord monitoring. Additional measures, which are also viable damage-control strategies, encompass the surveillance and release of intraspinal pressure by cerebrospinal fluid drainage as well as the increase and maintenance of systolic blood pressure and therefore available tissue oxygen [12]. However, delayed-onset spinal cord ischemia often remains beyond these measures [16]. Despite all efforts, SCI still appears in patients after TAAA repair, therefore necessitating effective treatment strategies for both immediate and delayed-onset SCI.

Depending on the treatment strategy of TAAA repair, pathomechanisms of spinal cord ischemia vary [12]. Replantation of intercostal arteries during open aortic repair can results in ischemia-reperfusion injury and subsequent inflammation, whereas permanent occlusion of afferent arteries in endovascular aortic repair leads to an ischemic insult or stroke of the affected spinal cord region [12]. However, both pathomechanisms result in exuberant inflammation of spinal cord tissue via significantly increased levels of proinflammatory cytokines—such as Interferon 1 beta, Interleukin 6, or Tumor necrosis factor alpha—activation of resident microglia and astrocytes, and recruitment and invasion of peripheral immune cells [21,22,23].

Extracorporeal shock wave therapy (SWT) has been in clinical use for lithotripsy or non-healing ulcers [24]. Within the last years, however, SWT has shown to exert beneficial effects in chronic ischemic tissues, such as chronic ischemic myocardium or muscle tissue [25,26]. In an animal model, direct postoperative SWT was also shown to ameliorate spinal cord ischemia [27]. Furthermore, it could be demonstrated that the activation of Toll-like receptor 3 (TLR3) and a subsequent modulation of inflammation was causal for these regenerative effects of SWT [27]. TLR3 is a receptor of the innate immune system that detects RNA and is not only present on immune cells but exists ubiquitously across human cell lines [28]. In the field of neuroscience, different in vivo studies showed that TLR3 activation via poly(I:C) in ischemic neuronal tissue leads to decreased neurological scarring after stroke or neonatal hypoxia [29]. These effects were also linked to a significant suppression of TLR4 through the TLR3 signaling pathway [30].

The aim of this study was to investigate whether pre- and postconditioning with either Toll-like receptor 3 agonist or shock wave therapy (SWT) could have depressing effects on inflammatory and apoptotic markers as surrogates for neuronal inflammation and degradation and therefore induce potential therapeutic benefits in SCI.

## 2. Materials and Methods

### 2.1. Cell Culture and Oxygen Glucose Deprivation

Cell culture was performed using SH-SY5Y (Sigma Aldrich/Merck, Darmstadt, Germany), a human neuroblastoma cell line, as a standard neuronal model. SH-SY5Y cells were cultured using DMEM 4.5 g High Glucose Medium (Pan Biotech, Aidenbach, Germany) containing 10% fetal calf serum and 1% penicillin–streptomycin–glutamine, split at a confluence of 70–90% and used until passage 22. Prior to hypoxia initiation, DMEM 4.5 g High Glucose Medium was replaced with DMEM 1 g Low Glucose Medium (Pan Biotech, Aidenbach, Germany). Afterwards, hypoxia was induced at normothermic conditions using a common hypoxia chamber, as previously described [31]. Duration of hypoxia varied according to experimental settings between two to twelve hours. After the termination of hypoxia, cell culture medium was changed back to DMEM 4.5 g High Glucose Medium for reoxygenation, and cell culture wells were randomly assigned to different experimental groups.

In a first set of experiments, genetic expression of proinflammatory cytokines was analyzed following two hours of OGD to study short-term effects of spinal cord ischemia, whereas subsequent experiments investigated the long-term effects of hypoxia after 12 h of OGD. 

### 2.2. Pre- and Postconditioning

To investigate different mechanisms of possible new treatment or preventive strategies for spinal cord ischemia and to compare isolated Toll-like receptor 3 stimulation to SWT, experiments with pre- and postconditioning of SH-SY5Y cells were performed. Cells were preconditioned either applying poly(I:C) (InvivoGen, San Diego, CA, USA), a TLR3 agonist dissolved in endotoxin-free physiological water, at a concentration of 100 ng/mL one hour before the initiation of oxygen glucose deprivation (OGD) or SWT with 200 impulses per well (energy flux density = 0.08 mJ/mm^2^, frequency = 3 Hz) using the Orthogold device with applicator CG050-P (TRT LLC, Tissue Regeneration Technologies, Woodstock, GA, USA). In postconditioning experiments, cells received poly(I:C) treatment or SWT, as described above, immediately after OGD at the beginning of reoxygenation, whereas control groups only received OGD without treatment.

### 2.3. Transfection/Small Interfering RNA (siRNA)

Transient transfection of SH-SY5Y cells to induce a temporary TLR3 knock-down was achieved using siRNA for TLR3 (Santa Cruz Biotechnology, Dallas, TX, USA) or AllStars Negative Control siRNA (scrambled siRNA) (QIAGEN, Hilden, Germany) as transfection control. DharmaFect 1 (Dharmacon, Horizon Discovery, Cambridge, UK) was used as transfection reagent according to the manufacturer’s protocol. Transfection efficacy was determined after 26 h, 30 h, and 48 h post transfection by Real-Time PCR (RT-PCR). Following successful transfection (Supplementary Materials, Figures S1 and S2), SH-SY5Y cells underwent OGD and pre-and postconditioning as described above.

### 2.4. Quantitative Real-Time PCR (qRT-PCR)

Cytokine expression was measured using qRT-PCR. Monarch Total RNA Miniprep Kit (New England Biolabs, Ipswich, MA, USA) was used according to the manufacturer’s protocol for cell harvesting and total RNA isolation and LunaScript RT SuperMix Kit (New England Biolabs, Ipswich, MA, USA) for cDNA transcription. qRT-PCR was performed using Luna Master Mix (New England Biolabs, Ipswich, MA, USA) according to the manufacturer’s protocol. Primers (see Supplementary Table S1) were designed using Primer Express Software (Version 3; Applied Biosystems; Life Technologies, Thermo Fisher Scientific, Waltham, MA, USA). qRT-PCR Amplification consisted of a two-step polymerase chain reaction (40 cycles: 1 min denaturation at 95 °C and 30 sec annealing/extension at 60 °C). Specific gene expression was normalized to GAPDH as housekeeping gene, and relative gene expression was calculated by the 2^−ΔΔCt^ method. Double determinations were used for calculation of mean Ct values. If Ct values exceeded 40 cycles, samples were categorized negative. Human primers used for PCR analysis can be found in Supplementary Materials, Table S1.

### 2.5. Western Blot

Western blot was used for apoptosis as well as signaling pathway analysis. Following the cell harvest using RIPA Buffer, protein samples were quantified using the Pierce BCA Protein Assay Kit (Thermo Scientific, Waltham, MA, USA) and stabilized with Laemmli SDS sample buffer (Alfa Aesar, Ward Hill, MA, USA). Gel electrophoresis and blotting were performed as previously described [32]. Ponceau dye was applied after blotting for quality control, followed by either 5% BSA or 5% non-fat milk as blocking solution. Primary antibodies (Supplementary Table S2) were incubated overnight at 4 °C, and secondary antibodies (polyclonal goat anti-rabbit and anti-mouse, both Dako, Glostrup, Denmark, and donkey anti-goat, Santa Cruz Technology, Dallas, CA, USA) were incubated for 1 h at room temperature. Amersham ECL Prime Western Blotting Detection Reagents (GE Healthcare, Chicago, IL, USA) were used for protein detection, and blots were scanned using the Bio-Rad ChemiDoc MP imaging system. Semiquantitative analysis of Western blots was performed using ImageJ. List of primary antibodies used for Western blotting can be found in Supplementary Materials, Table S2.

### 2.6. Statistical Analysis

Results are expressed as mean ± standard error of mean (SEM). Results were analyzed for Gaussian distribution, and statistical comparisons between two groups were performed using either Student’s *t*-tests or Mann–Whitney tests as appropriate. Multiple groups were analyzed by one-way ANOVA with Tukey post hoc analysis to determine statistical significance. Probability values < 0.05 were considered statistically significant and marked with stars. Statistical calculations were performed using GraphPad Prism 8 (GraphPad Inc., LaJolla, CA, USA).

## 3. Results

### 3.1. Postconditioning of SH-SY5Y Cells Using Poly(I:C) or SWT Causes a Differentiated Genetic Regulation of Hypoxia Inducible Factor 1 Alpha, Proinflammatory Cytokines, TLR3, and Its Downstream Signaling

**Figure 2:**
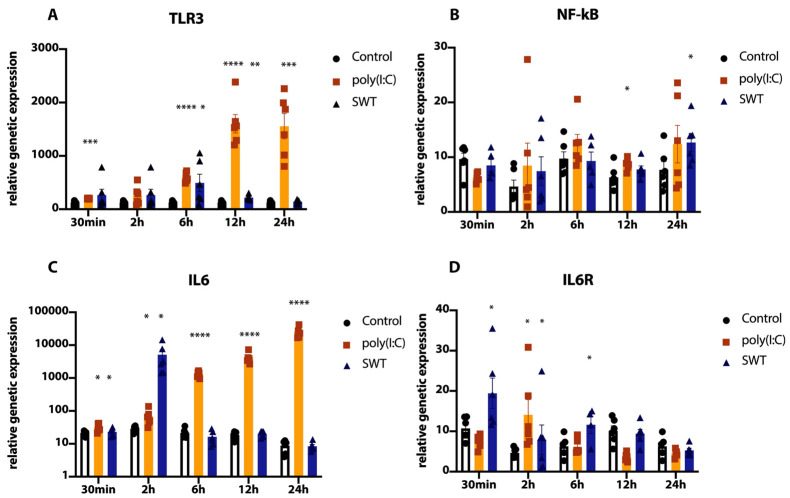
(**A**) Significant, linear increase of TLR3 genetic expression after postconditioning with poly(I:C). Comparatively lower peak of genetic expression at 6 h after treatment in the SWT group. **(B**) NF-kB expression demonstrates a biphasic pattern of genetic expression and no significant peak. (**C**) IL6 expression levels follow those of TLR3 with a linear increase in the poly(I:C) group and monophasic profile in the SWT group, reaching its peak at 2 h. (**D**) IL6R expression peaks at 30 min in the SWT group and at 2 h in the poly(I:C) group, followed by subsequent plummeting of mRNA levels in both groups. * *p* < 0.05; ** *p* < 0.01; *** *p* < 0.001; **** *p* < 0.0001.

**Figure 3:**
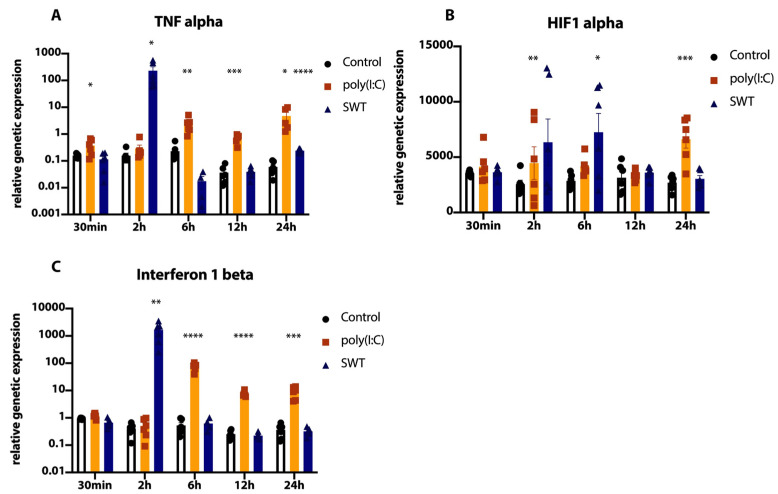
(**A**) SWT group displays a significant peak of TNF alpha mRNA levels at 2 h, whereas poly(I:C) reveals a biphasic pattern of genetic upregulation. (**B**) HIF1 alpha expression peaks in the SWT group 6 h after postconditioning. The poly(I:C) group presents a more biphasic shape of HIF1alpha mRNA levels, peaking at 24 h. (**C**) Interferon 1 beta mRNA levels peaking at 2 h in the SWT group while presenting with an expression pattern similar to TNF alpha. Significantly elevated mRNA levels at later time points in the poly(I:C) group. * *p* < 0.05; ** *p* < 0.01; *** *p* < 0.001; **** *p* < 0.0001.

To evaluate the effects of TLR3 activation either via the TLR3 agonist poly(I:C) or SWT in the setting of postconditioning on post-ischemic, reoxygenated neuronal cells, the genetic expression of TLR3, its downstream signaling, and proinflammatory cytokines along a defined time course were analyzed (Figure 2 and Figure 3).

The analysis of TLR3 demonstrated an opposite expression profile between the two groups, with a constant, nearly linear growth of TLR3 levels in the poly(I:C) group peaking at 24 h, whereas the SWT group showed a different dynamic by peaking at 6 h and, compared to poly(I:C), significantly (SWT 6 h vs. poly(I:C) 6 h, *p* < 0.0001) decreased levels of TLR3 (Figure 2A). On the contrary, the downstream transcription factor Nuclear Factor-kappa B (NF-kB) showed a biphasic course in both groups with no distinguishable peak (Figure 2B).

Moreover, the genetic expression of Interleukin 6 (IL-6) also demonstrated an almost linear rise in the poly(I:C) group, reaching its peak at 24 h, similar to TLR3 expression in the poly(I:C) group. Again, the SWT group reached its peak early at 2 h after treatment and plummeted again to reach IL-6 levels of the control group (Figure 2C). Concordantly, the SWT group reached IL6 receptor (IL6R) peak genetic expression at 30 min and decreased afterwards, whereas the poly(I:C) group peaked at 2 h but decreased more significantly (Figure 2D).

Differences between the genetic regulation of poly(I:C) and SWT post ischemia became apparent with regard to different cytokines. Similar to the course of the genetic expression of IL6 (Figure 2C), SWT showed a sharp peak in genetic expression of Tumor necrosis factor alpha (TNFalpha) (control 2 h vs. SWT 2 h, *p* = 0.0293) as well as Interferon 1 beta (IFN-b) (control 2 h vs. SWT 2 h, *p* = 0.0075) compared to controls at 2 h after reoxygenation and SWT (Figure 3A,C). The genetic expression of both cytokines decreased to levels of the control group afterwards (Figure 3A,C). In a similar dynamic, the SWT group reached peak genetic expression of Hypoxia inducible factor 1 alpha (HIF1 alpha) at 6 h and declined afterwards (Figure 3B). Contrary to this, poly(I:C) showed a more biphasic course of TNFalpha and HIF1 alpha genetic expression, demonstrating significantly elevated levels of genetic expression (control vs. poly(I:C) TNFalpha: *p* = 0.0005, control vs. poly(I:C) HIF1 alpha: *p* = 0.003) in later timepoints (Figure 3A,B). Similar to the dynamic of TNF alpha expression in the poly(I:C) group, IFN-b levels also remained significantly (control vs. poly(I:C) *p* < 0.0001) elevated in subsequent timepoints (Figure 3C).

### 3.2. Pre- and Postconditioning of poly(I:C) and SWT Lead to Diverging mRNA Levels of Cytokines and HIF1alpha

Cells were treated either before induction of hypoxia (preconditioning), after reoxygenation (postconditioning), or only with poly(I:C) continuously to investigate possible differences or similarities in genetic expression of cytokines in a time-dependent manner between these treatment strategies.

**Figure 4:**
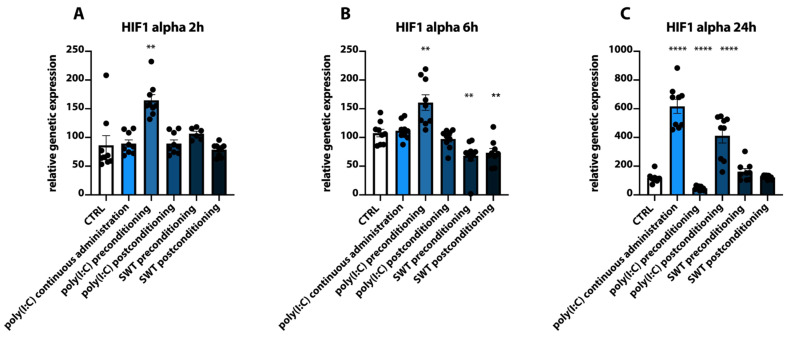
(**A**) At 2 h after reoxygenation, RT-PCR analysis reveals a significant increase in HIF1alpha expression after poly(I:C) preconditioning. (**B**) At 6h, both SWT pre- and postconditioning groups show significantly downregulated mRNA levels, while HIF1alpha expression remains significantly elevated in the poly(I:C) preconditioning group. (**C**) At 24 h, continuous administration of poly(I:C) as well as poly(I:C) postconditioning groups demonstrate significantly elevated levels of HIF1alpha mRNA, while the poly(I:C) preconditioning group is significantly downregulated. ** *p* < 0.01; **** *p* < 0.0001.

The analysis of mRNA levels of HIF1alpha revealed that its genetic expression was significantly upregulated at 2 h as well as 6 h after reoxygenation in the poly(I:C) preconditioning group compared to the control group (CTRL 2 h vs. poly(I:C) preconditioning 2 h *p* = 0.0010; CTRL 6 h vs. poly(I:C) preconditioning 6 h *p* = 0.0027) but significantly downregulated in this group at 24 h (*p* < 0.0001) (Figure 4A–C). On the other hand, continuous poly(I:C) administration as well as poly(I:C) postconditioning demonstrated significantly upregulated (CTRL 24 h vs. poly(I:C) postconditioning 24 h vs. poly(I:C) continuous *p* < 0.0001) HIF1alpha mRNA levels 24 h after reoxygenation with no significant differences in the genetic expression of HIF1alpha prior to this time point (Figure 4A–C). Contrary to these findings, SWT pre- and postconditioning groups only showed a significantly decreased HIF1 alpha expression 6 h after reoxygenation (CTRL 6 h vs. SWT preconditioning 6 h: *p* = 0.0027, CTRL 6 h vs. SWT postconditioning 6 h: *p* = 0.0030) and did not lead to significantly increased HIF1alpha levels at these timepoints (Figure 4A–C).

**Figure 5:**
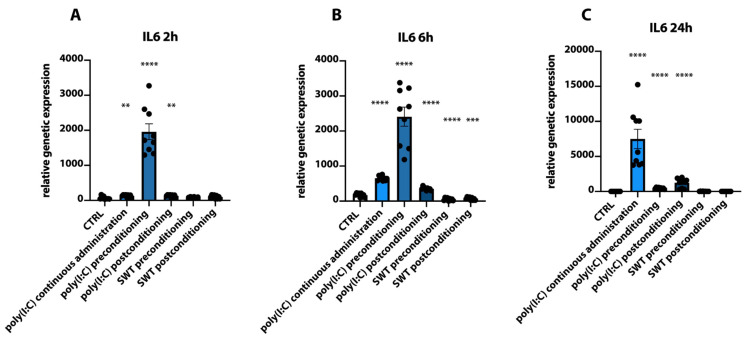
(**A**) Significantly upregulated mRNA levels of IL6 in the poly(I:C) preconditioning but also continuous administration and postconditioning groups at 2 h after hypoxia. (**B**) At 6 h, RT-PCR analysis demonstrates a similar expression profile of significantly upregulated poly(I:C) groups. In both SWT groups, IL6 mRNA levels are significantly decreased. (**C**) After 24 h, IL6 mRNA levels remain significantly elevated in the poly(I:C) continuous administration group. To a lesser extent, mRNA levels remain somewhat upregulated in poly(I:C) pre-and postconditioning groups. ** *p* < 0.01; *** *p* < 0.001; **** *p* < 0.0001.

When comparing the genetic expression of IL6 to HIF1alpha, similarities can be observed. Poly(I:C) preconditioning resulted in significantly increased levels of IL6 mRNA 2 h and 6 h after reoxygenation (CTRL 2 h vs. poly(I:C) preconditioning; CTRL 6 h vs. poly(I:C) preconditioning, *p* < 0.0001), whereas at 24 h, IL6 mRNA levels plummeted. Additionally, poly(I:C) postconditioning as well as continuous administration also showed significantly increased levels at 2 h (CTRL 2 h vs. poly(I:C) postconditioning 2 h, CTRL2 h vs. poly(I:C) continuous 2 h both: *p* = 0.0057) and 6 h (CTRL 6 h vs. poly(I:C) postconditioning 6 h, CTRL 6 h vs. poly(I:C) continuous 6 h both: *p* < 0.0001) compared to controls. However, both groups reached their peak genetic expression at 24 h. Similar to HIF1alpha, genetic expression of IL6 in SWT pre- and postconditioning groups did not demonstrate any significant upregulation at these timepoints (*p* = 0.2171 and *p* = 0.0595) and were indeed significantly (CTRL 6 h vs. SWT preconditioning 6 h: *p* < 0.0001; CTRL 6 h vs. SWT postconditioning 6 h: *p* = 0.0003) downregulated compared to controls at 6 h (Figure 5A–C).

**Figure 6:**
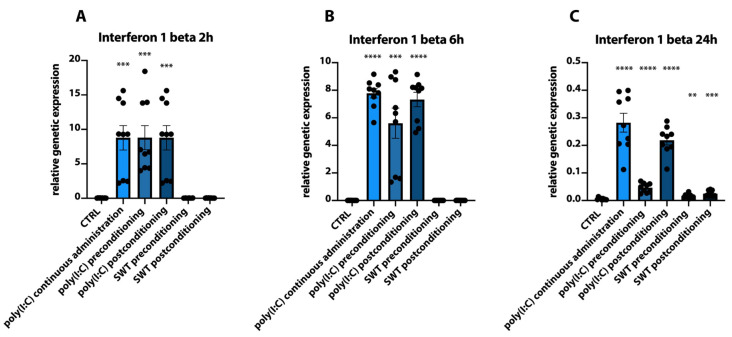
(**A**) Analysis of Interferon 1 beta (IFN1-b) expression at 2 h after hypoxia reveals significantly increased mRNA levels in the poly(I:C) groups, whereas SWT groups do not show significant upregulation. (**B**) Similar to (**A**), poly(I:C) groups demonstrate significantly increased IFN1-b expression at 6 h after hypoxia, whereas SWT groups remain at baseline. (**C**) IFN1-b mRNA levels decline but remain significantly elevated in Poly(I:C) groups, whereas SWT groups show a slight yet significant increase in IFN1-b mRNA levels. ** *p* < 0.01; *** *p* < 0.001; **** *p* < 0.0001.

Additionally, the genetic expression of Interferon 1 beta (IFN-b) painted a slightly different picture compared to the previously described HIF1 alpha and IL6. The poly(I:C) preconditioning group again showed significantly increased IFN-b levels at 2 h and 6 h (CTRL 2 h vs. poly(I:C) preconditioning 2 h; CTRL 6 h vs. poly(I:C) preconditioning 6 h, both: *p* = 0.0001), which decreased at 24 h. As opposed to the dynamics of HIF1 alpha and IL6, poly(I:C) postconditioning as well as continuous administration of poly(I:C) demonstrated significantly increased IFN-b levels as early as 2 h and 6 h after reoxygenation (CTRL 2 h vs. poly(I:C) continuous administration 2 h, CTRL 2 h vs. poly(I:C) postconditioning 2 h: both *p* = 0.0002, CTRL 6 h vs. poly(I:C) continuous administration 6 h; CTRL 6 h vs. poly(I:C) postconditioning 6 h: both *p* < 0.0001) and remained significantly elevated compared to controls at 24 h albeit at a lower level. SWT groups both did not show significantly upregulated levels of IFN-b at 2 h and 6 h and only slightly increased mRNA levels at 24 h at a comparatively low level of genetic expression (Figure 6A–C).

### 3.3. Poly(I:C) or SWT in Pre- and Postconditioning Leads to Temporary Suppression of TLR4 and Downstream Signaling

**Figure 7:**
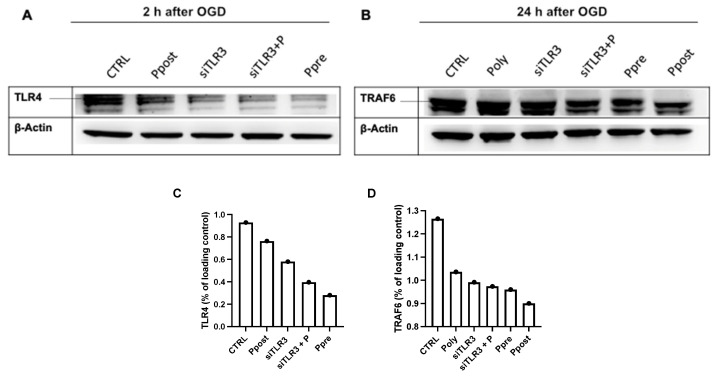
(**A**) 2 h after oxygen glucose deprivation (OGD), poly(I:C) preconditioning (Ppre) leads to significant suppression of TLR4 compared to controls (CTRL), as does poly(I:C) postconditioning (Ppost). Moreover, transient transfection prior to hypoxia results in significant TLR4 suppression (siTLR3, siTLR3+P) independent of poly(I:C) postconditioning after transfection and OGD (siTLR3+P); however, no effects are observed concerning TRAF6 in this group. (**B**) 24 h after OGD, TRAF6, a downstream signaling protein of TLR4, is suppressed in the poly(I:C) pre- and postconditioning groups, whereas continuous poly(I:C) administration (Poly) or transient transfection (siTLR3, siTLR3+P) does not show any differences in TRAF6 levels compared to controls. (**C**,**D**) Semiquantitative analysis of Western blots.

In ischemia-reperfusion injury, TLR3 stimulation has been shown to exert beneficial effects by suppressing TLR4 and its downstream signaling [30].To evaluate these findings in this experimental setting, Western blot analysis of poly(I:C) pre-and postconditioned cells of TLR4 and its downstream signaling molecule TRAF6 was performed (Figure 7A,B). The analysis indicated a higher suppression of TLR4 in the poly(I:C) preconditioning group compared to the postconditioning group. However, when comparing both groups to the control group (CTRL), TLR4 suppression was also apparent in the poly(I:C) postconditioning group. Besides, transient transfection with siTLR3 prior to hypoxia induction also showed a temporary suppression of TLR4 after OGD but not in TLR4 downstream signaling. Moreover, our Western blot analysis of TRAF6, a downstream effector molecule of the Myd88 pathway, demonstrated that both poly(I:C) pre- as well as postconditioning led to a significantly decreased presence of TRAF6 (Figure 7B).

**Figure 8:**
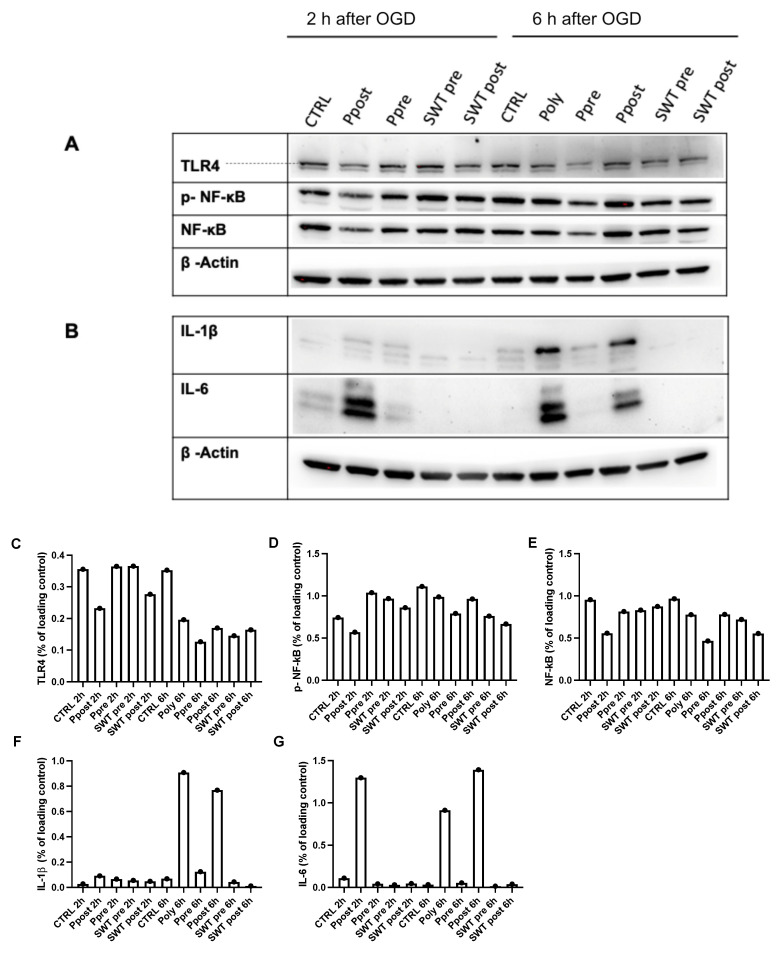
(**A**) At 2 h after OGD, TLR4 protein levels are significantly suppressed in the poly(I:C) postconditioning group (Ppost) but not in the SWT postconditioning group (SWT post) compared to controls (CTRL). Moreover, levels of active, phosphorylated NF-kB (p-NF-kB) are significantly decreased only in the poly(I:C) postconditioning group. At 6 h after OGD, poly(I:C) preconditioning (Ppre) as well as SWT pre- and postconditioning (SWT pre, SWT post) groups demonstrate TLR4 suppression. Ppre shows a significant downregulation of p-NF-kB compared to all other groups at this timepoint. (**B**) Western blot analysis 2 h and 6 h after OGD reveals significantly upregulated levels of proinflammatory cytokines Interleukin 1 beta (IL1-β) and IL-6 after poly(I:C) postconditioning (Ppost) and continuous poly(I:C) conditioning (Poly). At each interval, these cytokines remain significantly suppressed in both SWT groups. (**C**–**G**) Semiquantitative analysis of Western blots.

Apart from the Myd88 pathway, TLR4 activation can exert its inflammatory effects through the activation of NF-kB signaling [33]. Therefore, taking into account the aforementioned TLR4 suppression by TLR3 activation, Western blot analysis was performed (Figure 8). Analysis of pre- and postconditioning groups 2 h and 6 h after reoxygenation showed a suppression of TLR4 and its downstream NF-kB pathway in the poly(I:C) postconditioning group 2 h after OGD, whereas no changes were registered in the poly(I:C) preconditioning or either of the SWT groups at this timepoint. However, at 6 h after reoxygenation, TLR4 levels were significantly suppressed in the poly(I:C) preconditioning group and more subtly decreased in both SWT pre-and postconditioning groups but insignificantly distinctive between the poly(I:C) postconditioning and control groups (Figure 8A).

Moreover, proinflammatory cytokines Interleukin 1 beta (IL1-β) and IL6 were significantly upregulated in the poly(I:C) postconditioning group at 2 h and 6 h after reoxygenation, whereas all other groups did not produce significant concentrations of these cytokines compared to controls (Figure 8B).

### 3.4. Ischemic Pre- and Postconditioning via TLR3 Activation Leads to Decreased Apoptotic Markers Early after Reoxygenation

**Figure 9:**
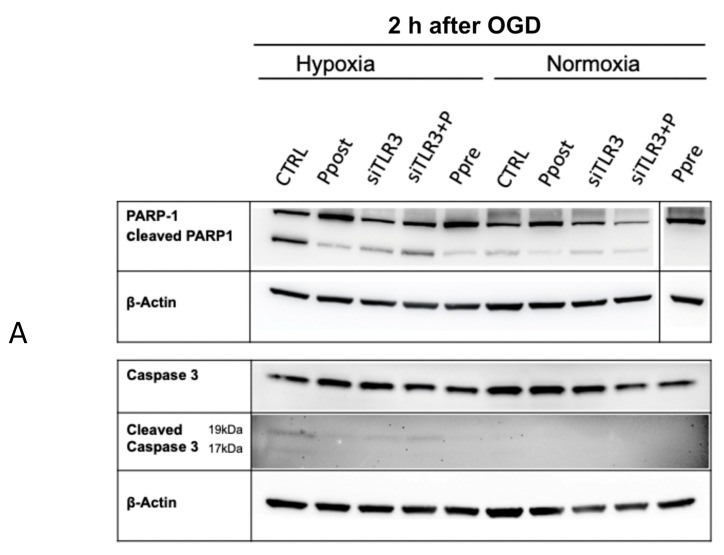

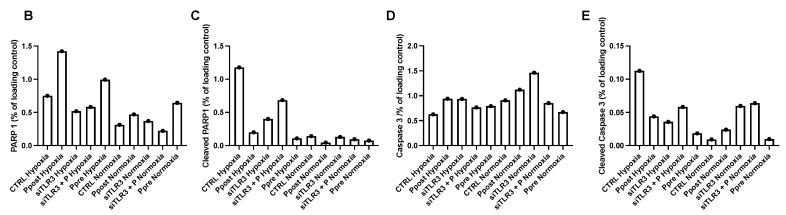
(**A**) Western blot analysis of apoptotic markers Caspase 3 and PARP-1 reveals significant downregulation of their activated, cleaved forms in the poly(I:C) postconditioning (Ppost) and preconditioning (Ppre) groups compared to controls (CTRL). Moreover, transient transfection prior to OGD (siTLR3, siTLR3+P) with post-hypoxic poly(I:C) stimulation (siTLR3+P) effectuates higher levels of cleaved apoptotic markers compared to Ppost and Ppre. No such differences were observed under normoxic conditions. (**B**–**E**) Semiquantitative analysis of Western blot.

In the setting of spinal cord ischemia, both acute ischemia as well as ischemia-reperfusion injury can cause neuronal apoptosis and can therefore lead to neurological deficits, such as para- or tetraplegia. As well-established indicators for apoptosis, we examined Caspase 3 and poly(ADP-ribose) polymerase1 (PARP-1) and their active (cleaved) forms in Western blot assays. Active (cleaved) forms of these proteins indicate the induction of apoptosis in these samples. In the setting of ischemic-reperfusion injury (hypoxia, 2 h after OGD), poly(I:C) pre- and postconditioning lead to significantly decreased levels of cleaved PARP-1 as well as cleaved Caspase 3 (Figure 9). Under normoxic conditions, no differences in the protein level of cleaved Caspase 3 could be observed, and the reduction of cleaved PARP1 by poly(I:C) pre- or postconditioning on a protein level was more subtle compared to hypoxic conditions.

In an effort to exclude possible confounding mechanisms behind these findings, TLR3 was temporarily knocked down (siTLR3, siTLR3+P) before hypoxia induction and postconditioned with poly(I:C) after reoxygenation (siTLR3+P). When comparing these groups to controls (CTRL), no significant differences could be observed, indicating that TLR3 activation is essential in the demonstration of this observed antiapoptotic effect (Figure 9).

**Figure 10:**
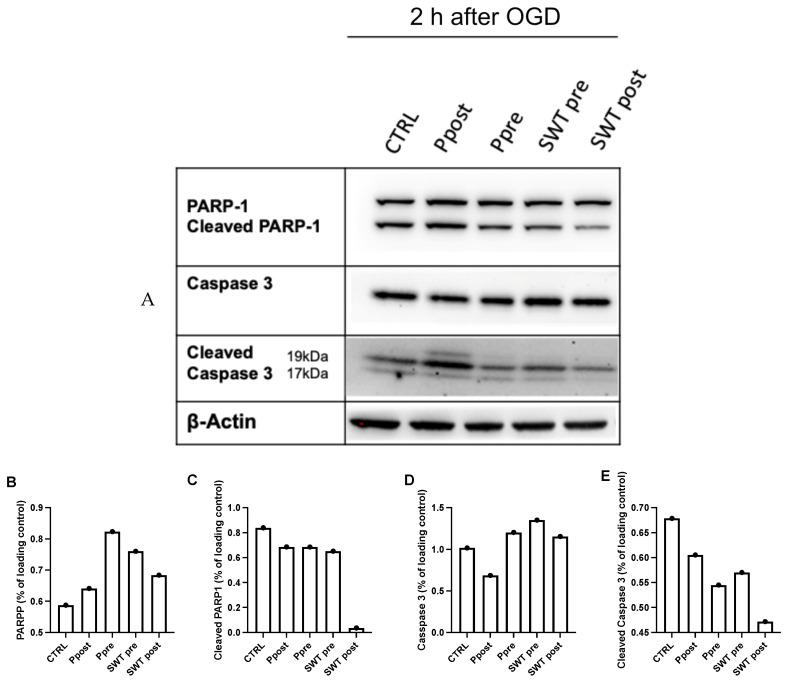
(**A**) Reduction of cleaved PARP-1 and cleaved Caspase 3 reaches its maximum in the SWT postconditioning group (SWT post). Levels of apoptotic markers in the SWT and poly(I:C) preconditioning groups (Ppre) are similar. (**B**–**E**) Semiquantitative analysis of Western blots.

Apart from pharmaceutical stimuli, SWT pre- and postconditioning groups also showed a decrease in the active, cleaved forms of Caspase 3 and PARP1, further emphasizing the role of TLR3 activation in decreasing neuronal apoptosis in the setting of ischemic neuronal injury, as has been established in earlier works [27]. However, when comparing SWT pre- and postconditioning, the latter seemed more effective in reducing levels of apoptotic markers and might therefore be a more efficient approach of SWT administration in spinal cord ischemia (Figure 10).

## 4. Discussion

Our results show that TLR3 activation causes significant alterations in post-ischemic neurons when achieved either pharmacologically via poly(I:C) or physically via SWT. Specifically, both procedures resulted in a shift in the genetic expression of (pro)inflammatory cytokines. In the poly(I:C) group, modulation of the inflammatory reaction turned out to be more pronounced and sustained, as we demonstrated a significant and steady increase of pro- but also anti-inflammatory cytokines, namely IL6, TNFalpha, and IFN-b. On the contrary, SWT induced more short-lived, singular peaks in genetic transcription. These findings might be a result of different modes and intensities of TLR3 activation, as SWT has been shown to exert its TLR3 activation indirectly via the cellular release of exosomes, whereas poly(I:C) is a direct TLR3 agonist [34].

Through cytokine analysis of pre- and postconditioned in vitro cells, groups treated with poly(I:C), especially those that underwent preconditioning, exerted more modulatory effects compared to the groups treated with SWT and control groups. These effects turned out similar across the analyzed cytokines as well as HIF1 alpha.

Regarding the modulation of inflammation following hypoxia, our experiments indicate stronger evidence for such modulation through poly(I:C) preconditioning compared to SWT. Particularly, the observed, long-lasting upregulation of IFN-b in our poly(I:C) groups suggests a more sustained modulation of inflammation. This implies a suppression of chronic inflammation, which may subsequently reduce long-term tissue damage and improve neurological outcomes. Moreover, the concomitant suppression of TLR4 and its signaling cascade might exert additional beneficial effects in the modulation of inflammation.

Supporting these arguments, both poly(I:C) pre- and postconditioning as well as SWT postconditioning resulted in a significant reduction of apoptotic markers, whereas anti-apoptotic effects of SWT preconditioning were less pronounced. These results might hint at possible beneficial effects by reducing neuronal apoptosis and therefore potentially decreasing the neurological impact of hypoxia.

To date, we cannot comprehensively evaluate the significance of these findings in a clinical setting of spinal cord ischemia. While clinical applications of poly(I:C), i.e., as part of peri-interventional conditioning strategies, are improbable due to its toxic effects in vivo, it seems that the pharmacological barrier for the clinical application of a TLR3 agonists has been surpassed: currently, a TLR3 agonist is being investigated in multiple phase II trials as a new oncological immunotherapy for the treatment of different cancers, such as melanoma [21]. Therefore, initial efforts to draft therapeutic or preventive approaches using direct TLR3 agonists or other immunomodulatory therapies might be in reach. Nonetheless, our findings indicate that SWT might also be a reasonable candidate for mitigating spinal cord ischemia. While an ongoing RCT will deliver further insights into the efficacy of cardiac SWT, its clinical application might be extensible to spinal cord ischemia as well [35].

Although TLR3 agonists seem to be a more promising treatment strategy for SCI when evaluated through our limited, in vitro scope, expectations might shift after both approaches have been evaluated in clinical trials. With more data available, currently unknown variables, including tissue and, respectively, blood–brain barrier penetration, adverse events, and clinical availability, might tip the balance towards a preference for SWT.

Apart from the particularities of future applications, our findings provide further insights into the molecular pathophysiology of spinal cord ischemia and highlight a potential for expanding the repertoire of effective treatment strategies.

Authors should discuss the results and how they can be interpreted from the perspective of previous studies and of the working hypotheses. The findings and their implications should be discussed in the broadest context possible. Future research directions may also be highlighted.

## Data Availability

Not applicable.

## Supplementary Materials

The following supporting information can be downloaded at: https://www.mdpi.com/article/10.3390/jcm11082115/s1, Table S1: Human primers used for PCR analysis; Table S2: Primary antibodies used for Western blotting. Figure S1: Transfection Control. Figure S2: Downstream signaling IRF3/NF-kB pathway after hypoxia.
