# Supplementary material for: Neuronal Pre- and Postconditioning via Toll-like Receptor 3 Agonist or Extracorporeal Shock Wave Therapy as New Treatment Strategies for Spinal Cord Ischemia: An In Vitro Study
Source: J Clin Med. 2022 Apr 11;11(8):2115. doi: 10.3390/jcm11082115 (PMC9027844; doi:10.3390/jcm11082115)
Supplement: Supplementary file 1 [file jcm-11-02115-s001.zip › jcm-1621496-supplementary.pdf]

Journal of Clinical Medicine

Supplementary Materials

Table S1. Human primers used for PCR analysis.

	forward	reverse
**TLR3**	5'-AGGAAAGGCTAGCAGTCATCC-3'	5'-TGCAGTCAGCAACTTCATGG-3'
**IL6**	5'-ATGATGGATGCTACCAAACTGG-3'	5'-TCTGAAGGACTCTGGCTTTGTC-3'
**TNF-α**	5'-TCATCAGTTCTATGGCCCAGAC-3'	5'-TTTGCTACGACGTGGGCTAC-3'
**HIF-1α**	5'-ATGTGACCATGAGGAAATGAGAG-3'	5'-TCGGCTAGTTAGGGTACACTTC-3'
**IFN-β1**	5'-TGAGAACCTCCTGGCTAATGTC-3'	5'-TTTTCAGGTGCAGACTGCTC-3'
**NF-κB**	5'-CCGCCTCTTCCTTCTCCAG-3'	5'-ACATTTGTTCAGGCCTTCCC-3'
**IL6 R**	5'-GTAGCCGAGGAGGAAGCATG-3'	5'-TCTCCTGGCAGACTGGTCAG-3'
**TLR4**	5'- CCCTGAGGCATTTAGGCAGCTA-3'	5'-AGGTAGAGAGGTGGCTTAGGCT-3'
**GAPDH**	5'-GGTGGTCTCCTCTGACTTCAACA-3'	5'-GTGGTCGTTGAGGGCAATG-3'

Table S2. Primary antibodies used for western blotting

Primary antibodies	Dilution
**TLR3** (Cell Signaling Technology, Danvers, MA, USA)	1:1000 in 5 % BSA-TBS-T
**TRIF** (TICAM-1)(Cell Signaling Technology, Danvers, MA, USA)	1:1000 in 5 % BSA-TBS-T
**TRAF6** (Cell Signaling Technology, Danvers, MA, USA)	1:1000 in 5 % BSA-TBS-T
**IRF3** (Cell Signaling Technology, Danvers, MA, USA)	1:1000 in 5 % BSA-TBS-T
**p-IRF3** (Cell Signaling Technology, Danvers, MA, USA)	1:500 in 5 % BSA-TBS-T
**Cleaved-Caspase 3** (Cell Signaling Technology, Danvers, MA, USA)	1:1000 in 5 % Milk-TBS-T
**Caspase 3** (Cell Signaling Technology, Danvers, MA, USA)	1:1000 in 5 % Milk-TBS-T
**PARP-1** (Cell Signaling Technology, Danvers, MA, USA)	1:1000 in 5 % Milk-TBS-T
**Caspase 8** (Cell Signaling Technology, Danvers, MA, USA)	1:1000 in 5 % BSA-TBS-T
**NF-κB** (Cell Signaling Technology, Danvers, MA, USA)	1:1000 in 5 % BSA-TBS-T
**p-NF-κB** (Cell Signaling Technology, Danvers, MA, USA)	1:1000 in 5 % BSA-TBS-T
**IL-1β** (Cell Signaling Technology, Danvers, MA, USA)	1:1000 in 5 % BSA-TBS-T
**TLR4** (Abcam, Cambridge, UK)	1:1000 in 5 % BSA-TBS-T
**IL-6** (Bio-Techne, Minneapolis, MI, USA)	1:1000 in 5 % BSA-TBS-T
**β-Actin** (Sigma-Aldrich, St. Luis, MI, USA)	1:10 000 in 5 % BSA-TBS-T

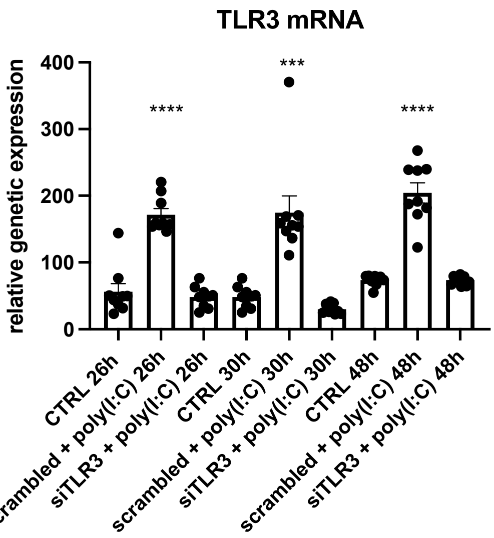

Figure S1. **Transfection Control.** RT-PCR analysis after transfection with siRNA TLR3 and scrambled siRNA demonstrates effectiveness of transfection 26h, 30h and 48 h after transfection. Stimulation of TLR3 vial poly(I:C) did not trigger any upregulation of genetic expression of TLR3 in the siTLR3 group, whereas scrambled siRNA group responded with a significant increase of genetic expression of TLR3, as it was expected.

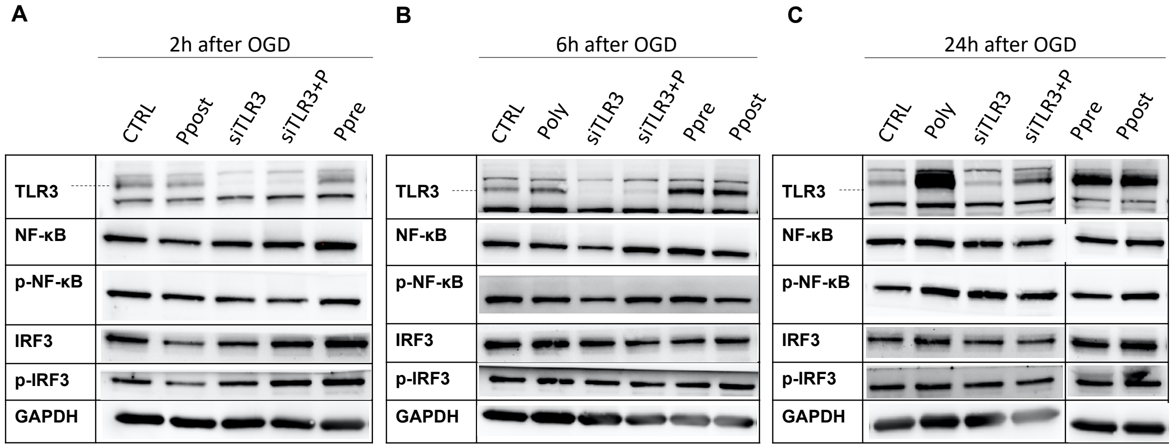

Figure S2. Downstream signaling IRF3/NF-kB pathway after hypoxia. (**A–C**) Analysis of TLR3 downstream signaling following hypoxia 2h, 6h and 24h after OGD. Western Blot analysis demonstrates downregulation of poly(I:C) postconditioning (Ppost) in TLR3 signaling, whereas poly(I:C) preconditioning (Ppre) leads to upregulation of TLR3 signaling 2h after OGD. At 6h and 24h these differences were not apparent.
